# Supplementary material for: Quantification of the Influence of Citrate/Fe(II) Molar Ratio on Hydroxyl Radical Production and Pollutant Degradation during Fe(II)-Catalyzed O2 and H2O2 Oxidation Processes
Source: Int J Environ Res Public Health. 2022 Oct 10;19(19):12977. doi: 10.3390/ijerph191912977 (PMC9566542; doi:10.3390/ijerph191912977)
Supplement: Supplementary file 1 [file ijerph-19-12977-s001.zip › ijerph-1949865-supplementary.pdf]

Supporting Information for

Quantification of the influence of citrate/Fe(II) molar ratio on hydroxyl radical production and pollutant degradation during Fe(II)-catalyzed O_2 and H_2O_2 oxidation processes

Bingbing Hu [a], Peng Zhang [a, b, *], Hui Liu [b], Songhu Yuan [a, b]

[a] State Key Laboratory of Biogeology and Environmental Geology, China University of Geosciences, 68 Jincheng Street, East Lake High-Tech Development Zone, Wuhan, Hubei, 430078, P. R. China

[b] Hubei Key Laboratory of Yangtze Catchment Environmental Aquatic Science, School of Environmental Studies, China University of Geosciences, 68 Jincheng Street, East Lake High-Tech Development Zone, Wuhan, Hubei, 430078, P. R. China

*E-mail: zhangpeng@cug.edu.cn (P. Zhang);

10 Pages, 3 Text, 4 Figures

Section S1: Descriptions of reactions and associated rate constants for kinetic modeling

In Table 1 in the main text, reactions A1–A4 describe the oxidation of inorganic $Fe(II)_{dis}$ by O_2, $•O_2^-$, H_2O_2 and •OH, reaction A5 describes the reduction of inorganic $Fe(III)_{dis}$ by $•O_2^-$, reactions A6–A7 describe the hydrolysis and precipitate of inorganic $Fe(III)_{dis}$, reactions A8–A9 describe the absorption and desorption of inorganic $Fe(II)_{dis}$ on lepidocrocite surface, reactions A10–A13 describe the oxidation of $Fe(II)_{ad}$ by O_2, $•O_2^-$, H_2O_2 and •OH, reaction A14 describes the decomposition of H_2O_2 by lepidocrocite and reaction A15 describes the reduction of lepidocrocite by $•O_2^-$. Reaction B1 describes the reaction between benzoate and •OH. Reactions C1–C4 describe the complexation of inorganic $Fe(II)_{dis}$ and $Fe(III)_{dis}$ by citrate, reaction C5–C8 describe the oxidation of citrate complexed Fe(II) by O_2, $•O_2^-$, H_2O_2 and •OH, reaction C9 describes the decomposition of H_2O_2 by citrate complexed Fe(III), reaction C10 describes the reduction of citrate complexed Fe(III) by $•O_2^-$, and reactions C11–C12 describe the oxidation of citrate complexed Fe(III) and uncomplexed citrate by •OH.

In the kinetic modeling, the rate constants for the oxidation of inorganic $Fe(II)_{dis}$, $Fe(II)_{ad}$, complexed Fe(II) by O_2 and the oxidation of complexed Fe(II) by H_2O_2 were obtained likewise by Kintecus V5.50 through best fitting the measured concentrations of $Fe(II)_{dis}$, $Fe(III)_{dis}$ and •OH (Figs. 1, 3 and S1). Other rate constants were referred to literature. As shown in Table 1, the rate constant of inorganic $Fe(II)_{dis}$ oxidation by

O_2 was much lower than that of Fe(II)$_{ad}$ and citrate complexed Fe(II) oxidation by O_2, which was ascribed to the high reactivity of Fe(II)$_{ad}$ and citrate complexed Fe(II) [1,2]. The rate constant of citrate complexed Fe(II) oxidation by H_2O_2 was lower than that of inorganic Fe(II)$_{dis}$ oxidation by H_2O_2, which was consistent with the previous observation that the rate of H_2O_2 decomposition by citrate complexed Fe(II) decreased with increasing citrate/Fe molar ratio [3].

In the absence of citrate, H_2O_2 reacted completely with inorganic Fe(II)$_{dis}$, so the •OH yield from H_2O_2 decomposition by inorganic Fe(II)$_{dis}$ can be set as 2.1%, 1.8% and 0.7% for pH 6, 7 and 7.5, respectively. Because the fraction of citrate complexed Fe(II) in total Fe(II)$_{dis}$ was higher than 99% at pH 6–7.5 when the citrate concentration reached 500 μM (i.e., citrate/Fe(II) was 2, Fig. S4), H_2O_2 will be almost completely decomposed by citrate complexed Fe(II). For the sake of simplicity, we assumed that the •OH yields from H_2O_2 decomposition by citrate complexed Fe(II) were equal to the experimental measurements when citrate/Fe(II) molar ratio was 2. Thus, the •OH yield from H_2O_2 decomposition by citrate complexed Fe(II) can be roughly set to be 52.2%, 31.5% and 17.6% for pH 6, 7 and 7.5, respectively.

Section S2 Influence of citrate/Fe(II) molar ratio on •OH production from H_2O_2 decomposition

To further get insight into the role of citrate/Fe(II) molar ratio on •OH production, we estimated the competitive effect of inorganic Fe(II)$_{dis}$ and citrate complexed Fe(II)

on H_2O_2 decomposition to generate •OH. In Fe(II)-citrate system, the relative contribution (R) of inorganic $Fe(II)_{dis}$ and citrate complexed Fe(II) to H_2O_2 decomposition can be calculated by Eq. (S1).

$$R = \frac{C_{Fe(II)_i} k_{H_2O_2, Fe(II)_i}}{C_{Fe(II)_{inorganic}} k_{H_2O_2, Fe(II)_{inorganic}} + C_{Fe(II)_{complexed}} k_{H_2O_2, Fe(II)_{complexed}}} \quad (S1)$$

where $C_{Fe(II)i}$ is the concentration of inorganic $Fe(II)_{dis}$ or citrate complexed Fe(II) and $k_{H2O2,Fe(II)i}$ was the reaction rate constant of the inorganic $Fe(II)_{dis}$ or citrate complexed Fe(II) with H_2O_2.

When the concentration of H_2O_2 was lower than that of Fe(II), the yield of •OH (φ) from H_2O_2 decomposition in Fe(II)-citrate system can be calculated by Eq. (S2),

$$\varphi = \varphi_{Fe(II)_{inorganic}} R_{Fe(II)_{inorganic}} + \varphi_{Fe(II)_{complexed}} R_{Fe(II)_{complexed}} \quad (S2)$$

where $\varphi_{Fe(II)inorganic}$ and $\varphi_{Fe(II)complexed}$ were the •OH yield from H_2O_2 decomposition by inorganic $Fe(II)_{dis}$ and citrate complexed Fe(II) (e.g., Fe(II)-citrate$^-$), respectively; $R_{Fe(II)inorganic}$ and $R_{Fe(II)complexed}$ were the relative contribution of inorganic $Fe(II)_{dis}$ and citrate complexed Fe(II) to decompose H_2O_2, respectively.

Combining Eqs. (S1)–(S2) gives

$$\begin{aligned} \varphi = {} & \varphi_{Fe(II)_{inorganic}} \frac{C_{Fe(II)_{inorganic}} k_{H_2O_2, Fe(II)_{inorganic}}}{C_{Fe(II)_{inorganic}} k_{H_2O_2, Fe(II)_{inorganic}} + C_{Fe(II)_{complexed}} k_{H_2O_2, Fe(II)_{complexed}}} \\ & + \varphi_{Fe(II)_{complexed}} \frac{C_{Fe(II)_{complexed}} k_{H_2O_2, Fe(II)_{complexed}}}{C_{Fe(II)_{inorganic}} k_{H_2O_2, Fe(II)_{inorganic}} + C_{Fe(II)_{complexed}} k_{H_2O_2, Fe(II)_{complexed}}} \end{aligned} \quad (S3)$$

In Eq. (S3), the concentrations of inorganic $Fe(II)_{dis}$ and complexed Fe(II) can be obtained from speciation calculation (Fig. S4), the reaction rate constants of inorganic and citrate complexed Fe(II) with H_2O_2 were given in Table 1, and the values of

$\varphi_{Fe(II)inorganic}$ and $\varphi_{Fe(II)complexed}$ were given in Table 2.

Section S3 Influence of citrate on Fe(II) oxidation and $Fe(III)_{dis}$ precipitate

Previous studies have shown that Fe(II) oxidation and Fe(III) precipitate in the inorganic $Fe(II)_{dis}$ system can be described as the following processes [4,5]: inorganic $Fe(II)_{dis}$ was first oxidized by O_2 to generate inorganic $Fe(III)_{dis}$, and then inorganic $Fe(III)_{dis}$ was hydrolyzed to Fe(III) precipitates. The resulting Fe(III) precipitates may adsorb $Fe(II)_{dis}$ forming adsorbed Fe(II) ($Fe(II)_{ad}$). Because $Fe(II)_{ad}$ toward O_2 was more reactive than inorganic $Fe(II)_{dis}$, the formation of Fe(III) precipitates can significantly accelerate inorganic $Fe(II)_{dis}$ oxidation [6,7]. At pH 6, no Fe(III) precipitates were generated in the inorganic $Fe(II)_{dis}$ system, so the oxidation of $Fe(II)_{dis}$ controlled the total rate of Fe(II) oxidation (Fig. S1).

In the presence of citrate, the complexation of citrate resulted in the formation of $Fe(II)\text{-}citrate^-$ and Fe(III)-citrate and the fractions of these complexed Fe species increased with increasing citrate/Fe molar ratio (Fig. S4). Because $Fecitrate^-$ toward O_2 was more reactive than Fe^{2+} [8], the presence of citrate accelerated $Fe(II)_{dis}$ oxidation at pH 6 and the rate of $Fe(II)_{dis}$ oxidation increased with increasing citrate concentration (Fig. 3). At pH 7 and 7.5, abundant Fe(III) precipitates produced in the inorganic $Fe(II)_{dis}$ system (Fig. S1), so the oxidation of $Fe(II)_{ad}$ contributed mainly to Fe(II) oxidation. However, the addition of citrate impeded the formation of Fe(III) precipitates in Fe(II)-citrate system (Fig. 3e–f), thus inhibiting $Fe(II)_{ad}$ generation. At

pH 7 and 7.5, the negative effect due to inhibiting Fe(III) precipitates outcompeted the acceleration effect of citrate complexed Fe(II) formation, thus inhibiting Fe(II) oxidation (Fig. 3b–c). For Fe(III) precipitates, in the absence of citrate, the resulting Fe^{3+} can be first hydrolyzed to dissolved Fe(III) oxyhydroxides (e.g., $Fe(OH)_2^+$ and $Fe(OH)_{3(aq)}$) (Fig. S4), which then interacted with one another to form Fe(III) precipitates [9]. However, in the presence of citrate, the complexation of citrate resulted in the formation of Fe(III)-citrate^0 complex (Fig. S4). In addition, a previous study reported that the presence of citrate prevented the crystallization of inorganic $Fe(III)_{dis}$ when citrate/Fe molar ratio was higher than 0.1 [10]. In this study, the molar ratio of citrate to Fe reached 0.25–2, which was higher than the threshold value. These two causes may jointly inhibit the formation of Fe(III) precipitates at pH 7 and 7.5.

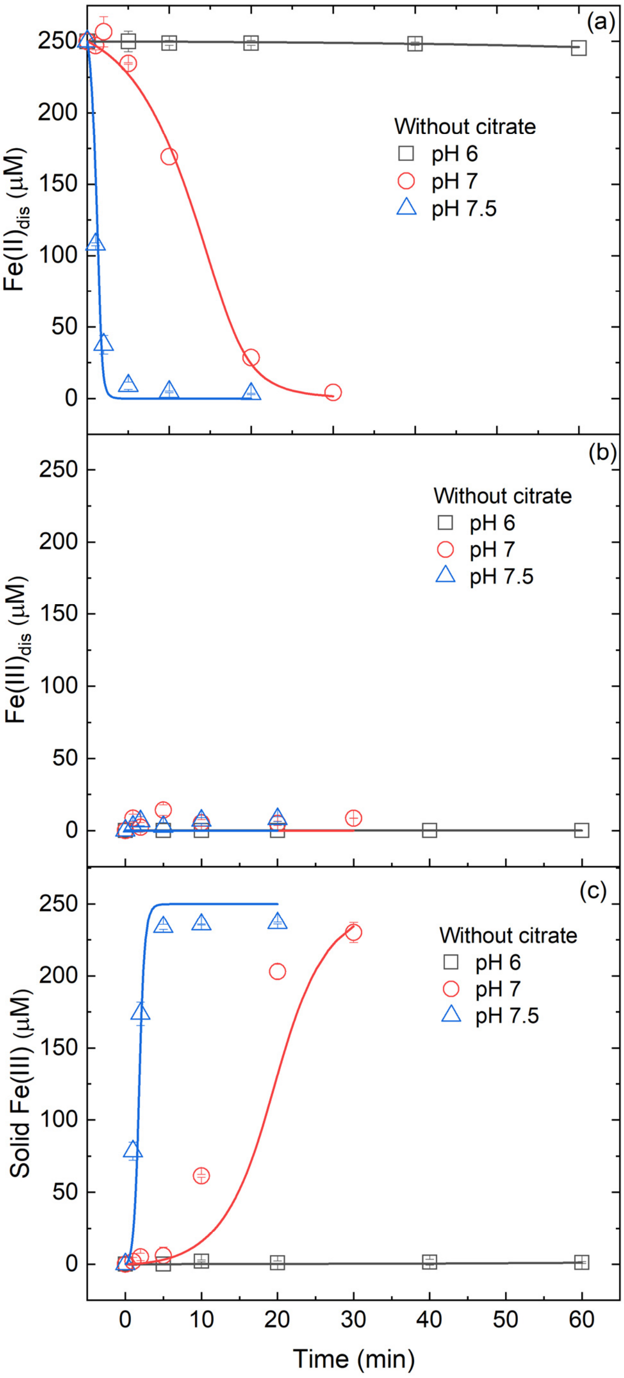

Figure S1. Effects of solution pH on (a) inorganic $Fe(II)_{dis}$, (b) inorganic $Fe(III)_{dis}$ and (c) solid Fe(III) production during oxidation. Initial conditions: 250 μM $Fe(II)_{dis}$, 20 mM benzoate and 10 mM buffer under oxic conditions. In panel (c), the concentration of solid Fe(III) was calculated using the difference between Fe_{total} and inorganic $Fe(III)_{dis}$ concentrations. Points are the experimental results and lines are the modelled

curves.

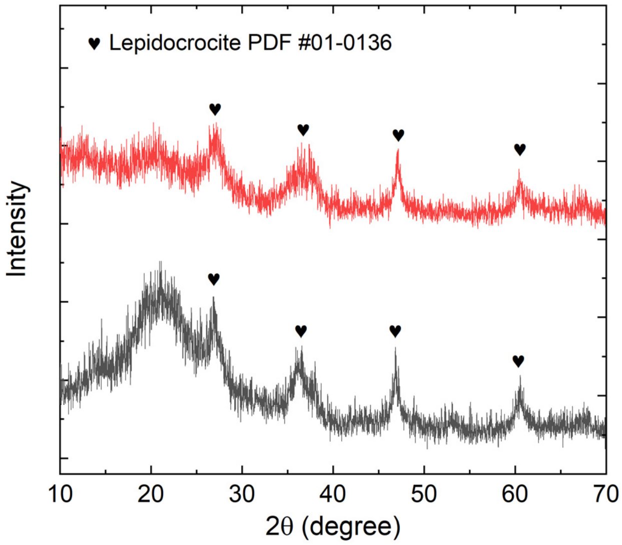

Figure S2. XRD patterns of Fe(III) precipitates. The main mineral compositions were detected by a D8-FOCUS X-ray diffractometer with Cu K radiation (Bruker AXS., Germany). The measurement was performed at 40 kV and 40 mA at the scanning step size of 0.01° and step time of 0.05 s.

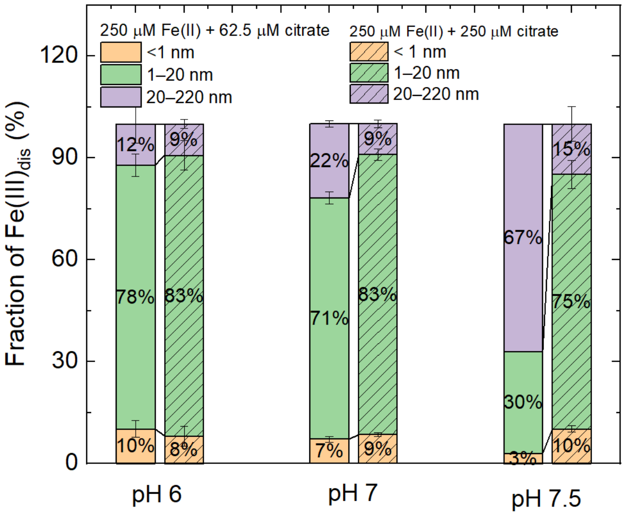

Figure S3. Percentage of Fe(III) concentration in different size fractions as a function

of solution pH and citrate dosage. The percentage of the y-axis means the concentration of Fe(III) in a certain size fraction to the total concentration of Fe(III) in the suspensions.

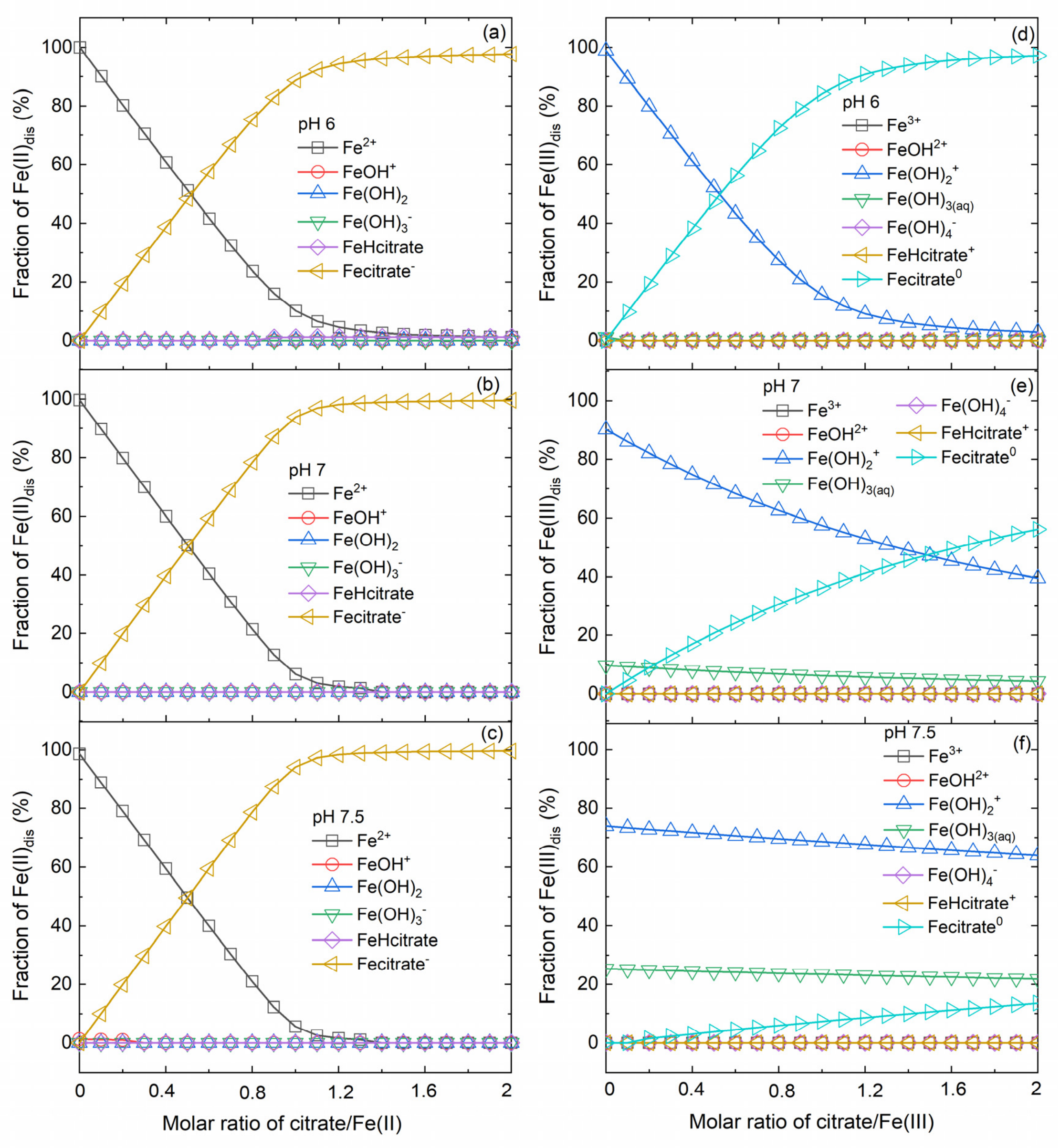

Figure S4. (a–c) Fe(II) and (d–f) Fe(III) species speciation distribution as a function of the molar ratio of citrate to Fe(II)/Fe(III). Initial conditions: variable solution pH and citrate concentration specified in panels (a–f) and 250 μM $Fe(II)_{dis}$/$Fe(III)_{dis}$.

Calculations were performed by Visual MINTEQ 3.1 [11]. The solid phase was not considered in both calculations.

References

1. Remucal, C.K.; Sedlak, D.L. The role of iron coordination in the production of reactive oxidants from ferrous iron oxidation by oxygen and hydrogen peroxide. In *Aquatic redox chemistry*; ACS Publications: 2011; pp. 177–197.
2. Jones, A.M.; Griffin, P.J.; Collins, R.N.; Waite, T.D. Ferrous iron oxidation under acidic conditions–The effect of ferric oxide surfaces. *Geochim. Cosmochim. Acta* **2014**, *145*, 1–12.
3. Lewis, S.; Lynch, A.; Bachas, L.; Hampson, S.; Ormsbee, L.; Bhattacharyya, D. Chelate-modified Fenton reaction for the degradation of trichloroethylene in aqueous and two-phase systems. *Environ. Eng. Sci.* **2009**, *26*, 849–859.
4. King, D.W.; Lounsbury, H.A.; Millero, F.J. Rates and mechanism of Fe(II) oxidation at nanomolar total iron concentrations. *Environ. Sci. Technol.* **1995**, *29*, 818–824.
5. Rose, A.L.; Waite, T.D. Kinetic model for Fe(II) oxidation in seawater in the absence and presence of natural organic matter. *Environ. Sci. Technol.* **2002**, *36*, 433–444.
6. Tamura, H.; Kawamura, S.; Hagayama, M. Acceleration of the oxidation of Fe^{2+} ions by Fe(III)-oxyhydroxides. *Corrosion Sci.* **1980**, *20*, 963–971.
7. Qian, A.; Yuan, S.; Xie, S.; Tong, M.; Zhang, P.; Zheng, Y. Oxidizing capacity of iron electrocoagulation systems for refractory organic contaminant transformation. *Environ. Sci. Technol.* **2019**, *53*, 12629–12638, doi:10.1021/acs.est.9b03754.
8. Zhang, P.; Van Cappellen, P.; Pi, K.; Yuan, S. Effects of riboflavin and desferrioxamine B on Fe(II) oxidation by O_2. *Fundamental Research* **2022**, *2*, 208–217, doi:https://doi.org/10.1016/j.fmre.2021.09.012.
9. Pham, A.N.; Rose, A.L.; Feitz, A.J.; Waite, T.D. Kinetics of Fe(III) precipitation in aqueous solutions at pH 6.0–9.5 and 25°C. *Geochim. Cosmochim. Acta* **2006**, *70*, 640–650, doi:10.1016/j.gca.2005.10.018.
10. Krishnamurti, G.; Huang, P. Influence of citrate on the kinetics of Fe(II) oxidation and the formation of iron oxyhydroxides. *Clay Clay Min.* **1991**, *39*, 28–34.
11. Gustafsson, J. Visual MINTEQ, Version 3.1 Division of land and water resources. *Royal Institute of Technology, Stockholm, Sweden* **2013**.
